# The Role of Gut Microbiota in the Pathogenesis of Glaucoma: Evidence from Bibliometric Analysis and Comprehensive Review

**DOI:** 10.3390/bioengineering11111063

**Published:** 2024-10-24

**Authors:** Zakir Ullah, Yuanyuan Tao, Amina Mehmood, Jufang Huang

**Affiliations:** 1 Department of Anatomy and Neurobiology, School of Basic Medical Sciences, Central South University, Changsha 410017, China; zakirullah.csu@gmail.com (Z.U.); taoyuanyuan1021@csu.edu.cn (Y.T.); 2 Taiwan International Graduate Program in Interdisciplinary Neuroscience, National Cheng Kung University and Academia Sinica, Taipei 115014, Taiwan; 3 Department of Microbiology and Immunology, College of Medicine, National Cheng Kung University, Tainan 701401, Taiwan; aminamehmood147@gmail.com

**Keywords:** glaucoma, gut microbiota, microbiota dysbiosis, gut-retina axis, inflammation, bibliometric analysis

## Abstract

The relationship between gut microbiota and glaucoma has garnered significant interest, with emerging evidence suggesting that gut dysbiosis, inflammation, and immune mechanisms may contribute to glaucoma pathogenesis. Understanding these interactions through the gut–retina axis offers new insights into disease progression and potential therapeutic options. This study combines bibliometric analysis and literature review to evaluate research trends and key research areas related to gut microbiota’s role in glaucoma. Our data were collected from the Web of Science Core Collection (WoSCC) and included the English original articles and reviews published between 1 January 2008, and 6 August 2024. Visual and statistical analyses were conducted using VOSviewer and CiteSpace. The analyses comprised 810 citations from leading journals, representing contributions from 23 countries/regions, 111 institutions, 40 journals, and 321 authors. Among the countries and regions involved, the USA and China were the leading contributors, publishing the most articles and being major research hubs. The *Experimental Eye Research* and *Investigative Ophthalmology & Visual Science* were the top journals in citation and co-citations that produced high-quality publications. The top 10 highly cited articles were published in high-ranking, top-quartile journals. The frequently occurring keywords were “glaucoma”, “microbiota”, “gut microbiota”, “inflammation”, “gut–retina axis”, and “probiotics”. Our study highlights the growing interest in the association between gut microbiota and glaucoma. It summarizes the possible ways gut microbiota dysbiosis, systemic and neuroinflammation, and autoimmune mechanisms contribute to glaucomatous pathogenesis. Future research should focus on mechanistic studies to elucidate the pathways linking gut microbiota to glaucoma development and progression.

## 1. Introduction

Glaucoma is a long-lasting eye illness characterized by the slow deterioration of retinal ganglion cells (RGCs) and their nerve. These cells connect with the optic nerve, and over time, this can result in complete vision loss. Globally, an estimated 80 million individuals have visual impairment caused by glaucoma [1]. The prevalence of glaucoma was around 64.3 million in 2013, increasing to 76 million by 2020, with predictions suggesting it could rise to 111.8 million by 2040 [2]. Potential factors that contribute to the loss of RGCs include increased intraocular pressure (IOP), oxidative stress, excitotoxicity, mitochondrial dysfunction, and neuroinflammation [3,4,5]. Glaucoma is closely linked to elevated IOP, as the aqueous humor applies greater force per unit area than usual on the inner surface of the eye. This may be due to obstructions in the eye’s trabecular meshwork (TM), which drains aqueous humor from the anterior chamber. Inadequate drainage leads to increased aqueous fluid, resulting in increased IOP. High IOP has been demonstrated to cause anomalies in the extracellular matrices of the retina and lamina cribrosa, leading to impaired optic nerve function and subsequently vision loss [1,6]. The development of glaucoma occurs gradually and causes a visual field loss until it reaches an advanced level without any noticeable symptoms [7]. To fully understand this complex pathogenesis, it is essential to have a comprehensive understanding of the aberrant functional alterations that occur at tissue, cellular, molecular, and genetic levels. The most common approach to treat glaucoma involves lowering IOP, typically through the use of topical medications, laser treatments, or surgical procedures [8]. However, topical therapies require frequent application and may lead to side effects [9]. Therefore, researchers are exploring alternative methods, including gene therapy [10], cell therapy [11], modulation of the gut microbiota [12], and neuroinflammation [13].

The gut is recognized as the body’s largest immunological organ, which is critical in maintaining immune balance [14]. Studies indicate that the human digestive system, crossing from the stomach to the distal colon, harbors over 10¹⁴ microorganisms [15]. The gut microbiota and its metabolites have been linked to various chronic conditions, including metabolic disorders, gastrointestinal diseases, cardiovascular issues, and neurodegenerative diseases [16,17]. Recently, the concept of a gut-eye axis has emerged [18], suggesting that signals originating from gut microbiota may trigger immune responses in the eye. Research has been initiated to uncover connections between gut microbiota and glaucoma. Nevertheless, the specific pathways through which the microbiome might trigger the onset of glaucoma remain uncertain [19]. Possible underlying mechanisms include the possibility that gut microbiota dysbiosis may allow harmful substances to the systemic circulation, triggering systematic inflammation that leads to neuroinflammation by disrupting the blood-brain barrier (BBB) and blood-retinal barrier (BRB) [20,21]. Similarly, dysbiosis in the ocular surface leads to overgrowth of pathogenic bacteria, resulting in intraocular inflammation [22]. These inflammations ultimately damage the RGCs and lead to glaucoma development [23]. Other studies have shown that the oral bacterial load in glaucoma patients is significantly higher than in those without, potentially exacerbating the disease’s severity and progression [24,25]. Many studies have linked the microbiome and glaucoma. However, comprehensive studies that critically explore current progress and future directions are still required.

Bibliometrics is an analytical method that employs statistical techniques to conduct quantitative and qualitative assessments of a large literature volume [26]. By extracting, analyzing, and visualizing bibliometric parameters, an objective perspective on the past progression of research within a specific field is provided, highlighting the potential possibilities for future studies [27]. In the medical domain, citation metrics are commonly used to pinpoint influential peer-reviewed publications and serve as a basis for evaluating journal rankings [28,29].

This review explores the existing body of literature to identify trends, gaps, and key research areas in this evolving field. The link of gut microbiota with glaucoma suggests that gut microbiota dysbiosis, inflammation, neuroinflammation, and autoimmune mechanisms may play critical roles in glaucoma pathogenesis, mediated through complex gut–retina interactions. Given this topic’s novelty and multidisciplinary nature, it is essential to integrate findings from diverse studies, ranging from molecular insights to therapeutic approaches. Our analysis provides a comprehensive overview of the current research scenario, highlighting the need for further studies to turn these insights into clinical applications.

## 2. Methodology

### 2.1. Data Retrieval and Collection

We conducted a detailed search for publications on gut microbiota and glaucoma using the following search formula (Table 1) on WoSCC (https://www.webofscience.com/wos/woscc/basic-search) (accessed on 6 August 2024).

The article trace time was from 1 January 2008 to 6 August 2024, focusing solely on English-language publications. Only original research and review articles were included. Initially, the authors (Z.U. and Y.T.) conducted independent searches within the WoSCC database and carefully evaluated the publications to ensure they were relevant to our research topic. Additionally, other authors reviewed and evaluated the inconsistencies until a consensus was achieved. All publications were exported as plain text files using the “Full Record and Cited References” format on 6 August 2024.

### 2.2. Data Analysis

The plain text files were imported into VOSviewer (version 1.6.11; Leiden University, Leiden, The Netherlands) to compile various bibliometric parameters and visualize them. Common parameters include average year of publication, citation count, average citation count, link, and cluster. The result can be exported in text format to Microsoft Excel 2010 (Redmond, WA, USA) for further analysis. VOSviewer emphasizes the scientific impact of countries/regions, institutions, journals, and authors in a particular field by presenting the collaboration networks among them and the strength of their collaborations. Furthermore, the co-occurrence analysis of author keywords helped identify research topics during specific times and uncover potential perspectives. CiteSpace was employed to scrutinize the most significant references and keywords in citations (Version 6.1. R1). In the VOSviewer plots, the size of nodes represents weight, which includes the number of documents, citation counts, or average publication year. The connections between nodes indicate a collaborative or co-occurrence relationship between them, and the thickness of these links signifies the strength of the collaboration. In the network visualization plot, node colors indicate various clusters, while in the overlay plot, these colors represent the average publication year. In CiteSpace, references are sorted according to the year in which the burst started, and a red bar chart represents the burst duration.

Based on a bibliometric analysis of the 48 articles, we identified key research areas pivotal to understanding this connection. The focus then narrowed to critical aspects, such as gut microbiota dysbiosis, inflammation, and autoimmune mechanisms, which are hypothesized to contribute to glaucomatous neurodegeneration. This approach provides a detailed discussion to clarify the potential association.

## 3. Bibliometric Analysis

### 3.1. General Data

**Figure 1:**
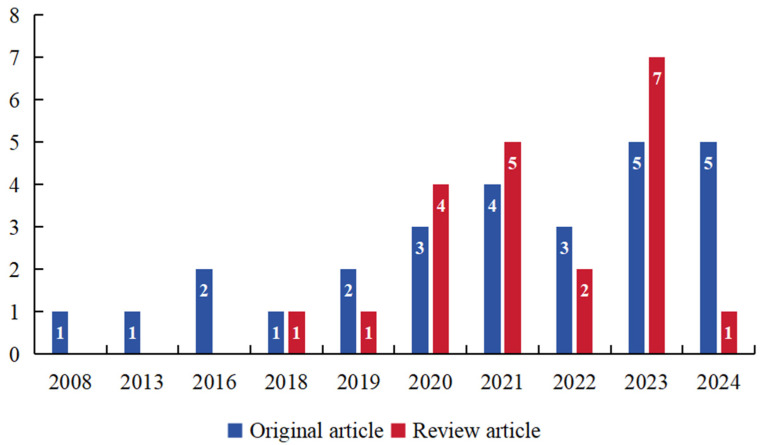
The overall annual publication trends.

A total of 50 articles published between 2008 and 2024 were sourced from the Web of Science Core Collection (WoSCC) database. The selection process was refined to include only English-language reviews and original research articles, resulting in a final analysis of 48 papers. Figure 1 illustrates the annual publication trends, showing the distribution of articles over the years. Since the data were gathered in August 2024, a continued increase in publications for 2024 is anticipated, following the patterns of earlier years. The publication volume grew consistently, with noticeable peaks in 2021 (n = 9) and 2023 (n = 12). The analysis comprised 810 citations from leading journals, including contributions from 23 countries/regions, 321 authors, 40 journals, and 111 institutions. The USA and China stood out as the major contributors to this field of research.

### 3.2. Analysis of the High-Productivity Countries or Regions

**Figure 2:**
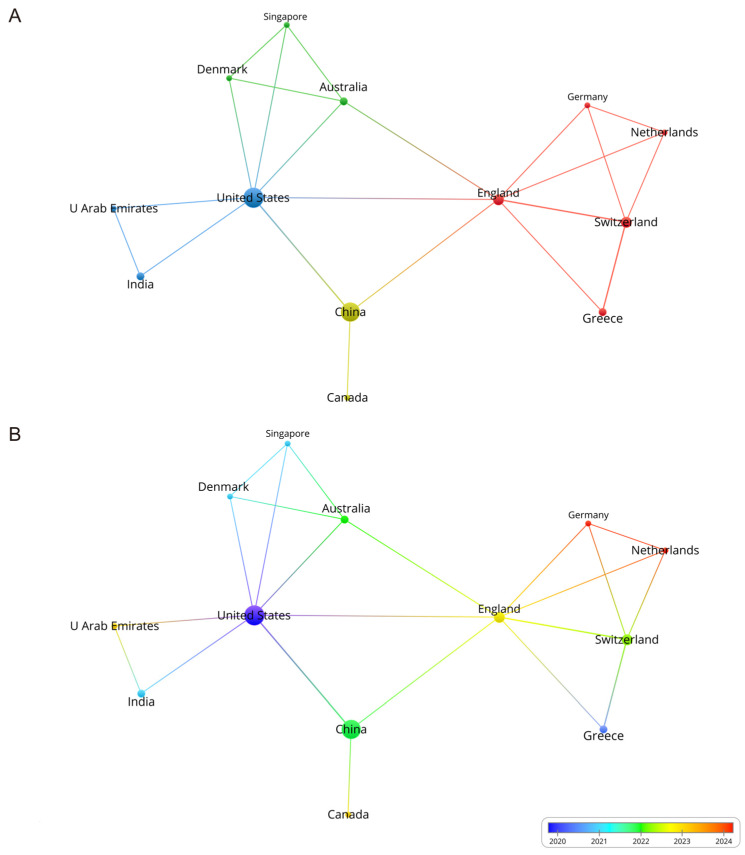
(**A**) The network visualization plot displays global research partnerships in gut microbiota and glaucoma research. The size of the node reflects the volume of publications. (**B**) The overlay visualization plot depicts the evolution of partnerships between countries/regions over time, with color indicating the average publication year.

Among the 23 countries or regions, 13 countries or regions have cooperation between them (Figure 2A). The United States ranked as the most prolific country, contributing 16 articles and 433 citations. China followed closely with 14 publications and 219 citations, while England published 4 articles with 35 citations. The USA and China are the pioneers in this field, as more than half of the articles in this analysis are from the USA and China, reflecting its continued commitment. The USA showed the highest level of collaboration with other leading countries, followed by China. The overlay plot offers valuable insights into the timeline of each institution’s contributions to this field, providing a detailed view of their ongoing cooperation. Early contributions are indicated in blue, while more recent ones are marked in red (Figure 2B).

### 3.3. Analysis of Prolific Institutions

**Figure 3:**
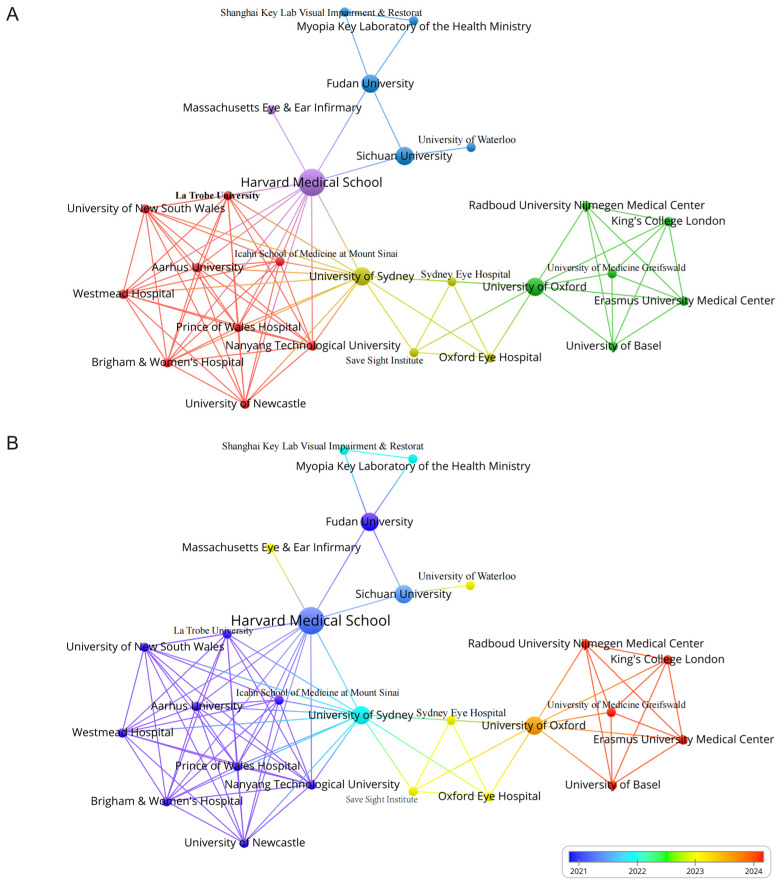
(**A**) The collaborative network plot illustrates the global research partnerships between institutions. The size of each circle corresponds to the institution’s publication output. (**B**) The overlay map displays institutional collaborations over time, with blue indicating earlier research activity and red representing more recent efforts.

A total of 111 institutions have contributed to the research on gut microbiota and glaucoma, with 26 of them actively engaging in collaborative partnerships. Table 2 shows the leading institutions in terms of the number of publications. Sun Yat Sen University has published five articles garnered 152 citations, followed by Harvard Medical School, which has three articles with 26 citations. It is worth highlighting that the University of Chicago has achieved a citation of 47 despite publishing only two publications. A cooperative relationship map (Figure 3A) has been constructed to illustrate the collaborative landscape. This visualization highlights the grouping of author affiliations and the strength of collaboration between institutions from 2008 to 2024. The University of Sydney exhibited the highest number of cooperative connections (n = 14), closely followed by Harvard Medical School (n = 13). Notably, research on gut microbiota and glaucoma has seen significant advancements in recent years (2021-2024), driven by the strong collaboration among these institutions and their concentrated efforts in this field. The overlay map (Figure 3B) provides valuable insights into each institution’s contributions timeline, offering a broad view of their sustained cooperation. Early-stage research is depicted in blue, while more recent work is marked in red.

### 3.4. Analysis of the Most Cited Journals

From 2008 to 2024, all the articles were published in 40 journals. The top five most prolific journals and their publication and citation are presented in Table 3. *Experimental Eye Research* (n = 3; 74 total citations), *International Journal of Molecular Sciences* (n = 3; 22 total citations), and *International Ophthalmology* (n = 3; 20 total citations) were the top journals in terms of publications. Regarding co-citations, *Investigative Ophthalmology & Visual Science* emerges at the top with 193 co-citations, followed by *PloS One* (113 co-citations) and *Scientific Reports* (102 co-citations).

### 3.5. Analysis of Keywords

**Figure 4:**
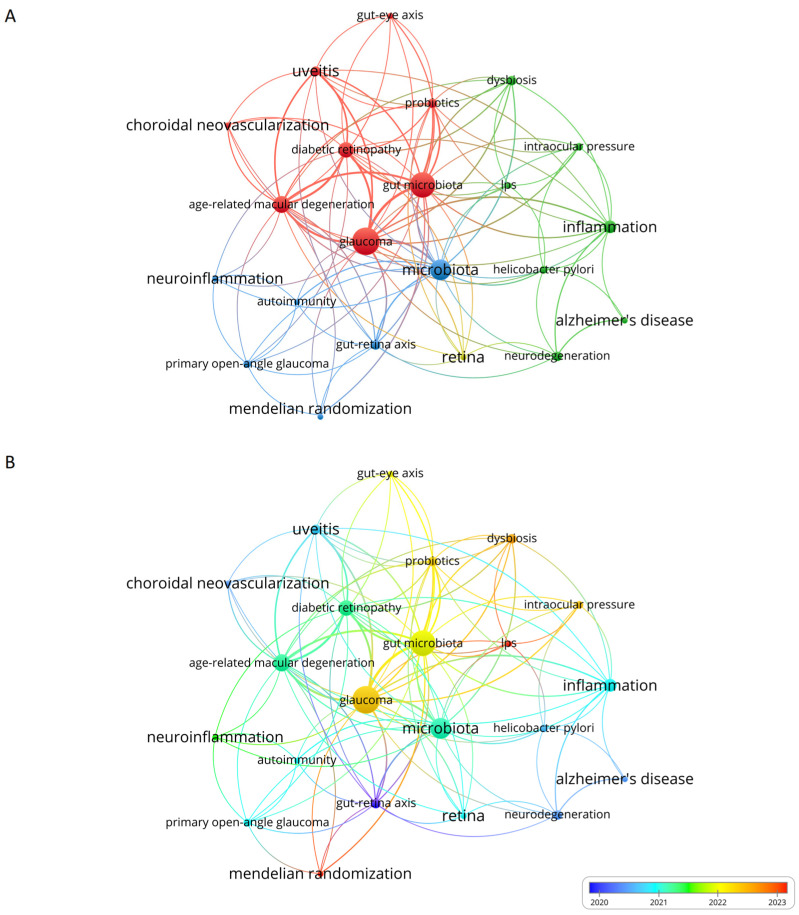
(**A**) The co-occurrence plot illustrates grouping the most used keywords. Keywords that appear together in the same document are linked, creating four clusters, each represented by different colored dots. (**B**) The overlay visualization map shows keywords over time, highlighting the initial research phase in blue and the subsequent research phase in red.

Assessing the co-occurrence of author keywords across different literature sources enabled us to identify and classify terms that occur frequently and delve into their interrelations. Utilizing VOSviewer, we recognized a total of 142 keywords, among which 22 were mentioned at least twice and were analyzed further (Figure 4A). The frequently occurring keywords were “glaucoma”, “microbiota”, “gut microbiota”, “inflammation”, “gut-retina axis”, and “probiotics”. A strong association was found among the following terms: “glaucoma”, “inflammation”, and “gut microbiota”, highlighting the critical role of microbiota-persuaded inflammation in the context of glaucoma [30,31,32]. Within the overlay visualization plot (Figure 4B), blue denotes the initial research phase, whereas red signifies the later phase of the study. In recent years, there has been an increased emphasis on “gut microbiota”, “dysbiosis”, “gut–eye axis”, “probiotics”, and “lps”.

### 3.6. Analysis of References

**Figure 5:**
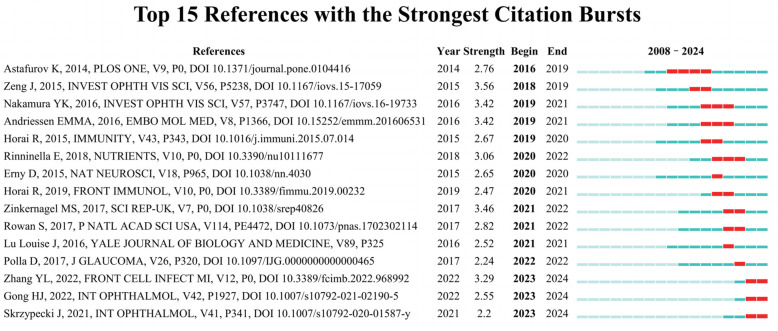
The 15 most frequently cited references exhibited the most robust citation bursts in research on gut microbiota and glaucoma from 2008 to 2024. “Year” represents the year the reference appears for the first time. “Begin” refers to the year of the beginning of the burst, and “end” refers to the year of the end of the burst. “Strength” represents the burst strength. The red line represents the specific diachronic stage when the reference becomes a hot topic of academic research [24,30,31,32,33,34,35,36,37,38,39,40,41,42,43].

When the burst duration is one year, there are 15 references exhibiting the most significant citation bursts (Figure 5). The article “The Relationship Between Helicobacter pylori Infection and Open-Angle Glaucoma: A Meta-Analysis” by J. Zeng, published in *Investigative Ophthalmology & Visual Science*, revealed the greatest burst strength, showing a value of 3.56. Additionally, three articles experienced citation bursts that ended in 2024, indicating that they have garnered increasing consideration recently.

## 4. Research Hotspots

This research employed bibliometric techniques to depict the existing studies on gut microbiota and glaucoma. The findings from our study show a consistent rise in the volume of publications as time progresses. This increasing trend reflects the growing importance and advancements in the field. Notably, this rise in publication volume may be linked to the increasing prevalence of glaucoma, as the WHO reported. It is estimated that the global number of individuals with glaucoma will reach 111.8 million by 2040, with a current count of at least 80 million affected individuals [44]. The top six most frequently occurring keywords in our bibliometric analysis were “glaucoma”, “microbiota”, “gut microbiota”, “inflammation”, “gut–retina axis”, and “probiotics”. In recent years, there has been an increased emphasis on “gut microbiota”, “dysbiosis”, “gut–eye axis”, and “probiotics”. These findings collectively indicate that researchers have made significant scientific developments and found new research areas for future studies. The bibliometric analysis conducted in this study makes the foundation for a targeted review exploring the role of gut microbiota in the pathogenesis of glaucoma. Significant research has been conducted to understand this connection; however, the exact mechanisms by which gut microbiota influence glaucoma remain largely unexplored. Addressing this knowledge gap is necessary for establishing a complete association between gut microbiota and glaucoma, which could open opportunities for novel therapeutic strategies. We explored a detailed analysis of the 48 articles identified through our bibliometric study, summarizing their findings to shed light on the role of gut microbiota in the pathogenesis of glaucoma. These articles focus on key areas such as the gut–retina axis, gut microbiota dysbiosis, inflammation, neuroinflammation, and autoimmunity, which significantly link gut health to glaucoma.

## 5. The Microbiota–Gut–Retina Axis

**Figure 6:**
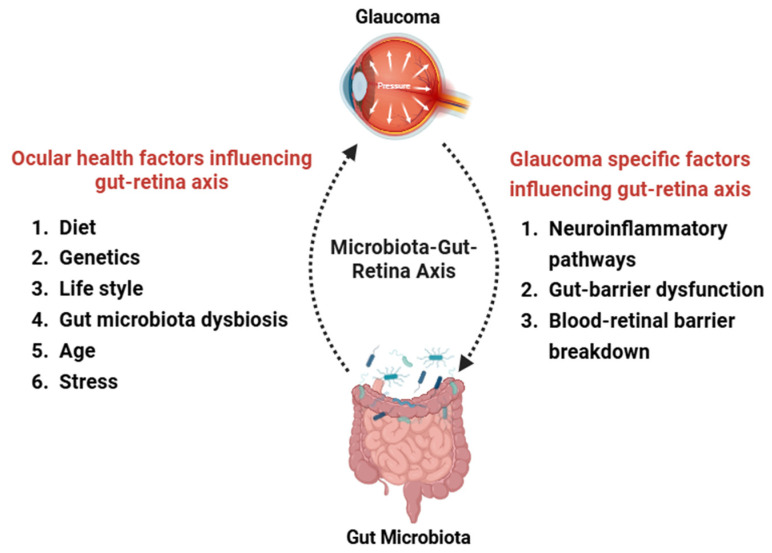
The figure shows the intricate relationship between gut microbiota and glaucoma, emphasizing both shared and glaucoma-specific factors that affect the gut–eye axis. Created using BioRender.com.

The “microbiota–gut–retina axis” plays a vital role in developing and progressing glaucoma and other eye disorders. The “microbiota–gut–retina axis” is considered crucial in the development and progression of glaucoma and other eye diseases like uveitis, age-related macular degeneration, and diabetic retinopathy (Figure 6) [45,46]. In this regard, maintaining gut homeostasis is essential for the protection of retinal health, as it regulates the host’s immune response and produces various anti-inflammatory compounds, including short-chain fatty acids (SCFAs), bacteriocins, secondary bile acids, indoles, and polyamines. However, recent studies indicate that disruptions in gut microbiota and their metabolites, particularly SCFAs, lead to gut obstruction, metabolic endotoxemia, systemic inflammation, and retinal impairment [30,47]. In this regard, the increased gut permeability and microbial translocation play a critical role in perpetuating systemic inflammation [48]. A sudden increase in IOP could disrupt the tight and adherent junctions of the retinal pigment epithelium (RPE) and ultimately affect the stability of the BRB in animal models of glaucoma [49], which is deemed a crucial step in the development of glaucoma [50]. Although this hypothesis lacks definitive evidence, it is mainly based on observations of T-cell infiltration in the retina in various animal models of glaucoma [51]. In dogs diagnosed with primary glaucoma, the damage to the RPE and the leakage of T cells and plasma proteins suggest disruption of the inner and outer blood-retinal barrier [52]. The progression of neurodegeneration in patients with glaucoma is a gradual process, and the disruption of the BRB is likely associated with prolonged inflammation in the retina [53]. A clinical study supports this finding as patients with primary open-angle glaucoma (POAG) exhibit flame-shaped hemorrhages near the edge of a damaged nerve fiber layer and atrophy of the choriocapillaris and RPE, indicating BRB dysfunction that may exacerbate neuronal damage [50]. The presence of markers of neurodegenerative diseases in the central nervous system, such as amyloid-beta (Aβ) and hyperphosphorylated tau (p-tau), has been found in the retina of patients with glaucoma [54]. These degenerative proteins trigger a localized inflammatory response and impair the function of the BRB by disrupting the tight junctions of the RPE and activating retinal microglia. This para-inflammatory condition leaves the retina more susceptible to infiltration by adaptive immune cells from peripheral areas [55].

Aging contributes to the “gut–eye axis” by compromising physical barriers, altering immune regulatory signals, and enhancing permeability. This occurs in the retina due to the damage of the protective tissue layers that help in the eye’s immune privilege and the intestines [48]. Additional elements influencing the gut–eye axis include epigenetic factors, dietary habits, and metabolites produced by the gut microbiome (Figure 6) [47]. The impact of diet on the gut–eye axis has been widely explored. Studies suggest intermittent fasting alters the gut microbiome, similar to effects seen in the gut–brain axis, and lowers risk factors for eye disorders [56,57].

## 6. Gut Microbiota Dysbiosis

Research on the importance of gut microbiota in retinal and ocular illnesses is in its early stages. Several studies have shown a possible connection between the gut microbiota and ocular diseases such as age-related macular degeneration, diabetic retinopathy, uveitis, and glaucoma [48,58,59,60,61,62,63]. In 2000, a link was first noticed between gut microbiota and glaucoma, with 88% of glaucoma patients found to have stomach *Helicobacter pylori* infection compared to 47% of controls [64]. More recent meta-analyses have also found an association between active *Helicobacter pylori* infection and primary open-angle glaucoma [65]. Treatment for *Helicobacter pylori* infection in POAG patients has reduced intraocular pressure and improved visual acuity. However, the exact nature of the relationship between *Helicobacter pylori* and glaucoma remains unclear [66,67].

Several studies in our bibliometric analysis explored the relationship between gut microbiota dysbiosis and their metabolic analysis with glaucoma. Gong et al. conducted a survey that compared the gut microbiota and blood metabolic profiles of POAG patients to healthy individuals. They found lower levels of *Escherichia coli* and *Megamonas* and higher levels of *Prevotellaceae* and *Enterobacteriaceae* in POAG patients. The metabolomic analysis showed a positive correlation between citric acid and *Megamonas*, while L-glutamyl-L-alanine, 3-methoxy-4-hydroxyphenylglycol, cholic acid glucuronide, and hypoxanthine showed a negative correlation [68]. Another study correlates variation in carbon-related metabolites with microbiota, suggesting they may harm RGCs [31]. Similarly, Zhang et al. found the same results that the glaucomatous rat model had different bacterial abundances (*Firmicutes*, *Verrucomicrobia*, *Romboutsia*, *Akkermansia,* and *Bacteroides*) compared to controls, with negative links observed with RGCs. Glutathione was negatively correlated with *Bacteroides* and *Firmicutes* and positively correlated with RGCs [32].

The glaucoma-specific metabolic SCFAs and the glaucoma-associated microbiota were revealed in a recent study through mass spectrometry, metagenomic, and 16S RNA sequencing. They found that the relative abundances of 299 genera significantly differed between the healthy and POAG groups. *Enterobacteriaceae*, *Eubacteriaceae*, *Clostridiaceae, Rikenellaceae*, *Bifidobacteriaceae*, *Ruminococcaceae*, *Prevotellaceae*, *Lachnospiraceae*, *Selenomonadaceae*, and *Bacteroidaceae* were abundant in POAG patients, while *Barnesiellaceae* were more abundant in healthy controls. Their finding suggests that the gut microbiota may contribute to the onset of glaucoma. At the same time, the gut–retinal axis may influence retinal microglia activation via retinal miRNAs [69].

Many studies have associated mitochondrial DNA (mtDNA) abnormalities with POAG. This association has also been linked with changes in the gut microbiota, specifically *Bacteroides* and *Prevotella* [70]. Increased mtDNA deletion has also been linked to increased cell death, which may increase the risk of glaucoma-related apoptosis in POAG patients [71]. Epigenetic modifications, such as irregular histone acetylation and deacetylation, could also contribute to RGC injury in glaucoma that can be triggered indirectly or directly by gut microbiota dysbiosis [72].

A preliminary study found that patients with glaucoma had significantly higher levels of trimethylamine, a toxic byproduct of gut bacteria [73]. They also found another gut bacteria byproduct, butyrate, decreasing the IOP in normotensive rats but not hypertensive rats [33]. Two independent European cohort studies (the United Kingdom Birth Cohort and the Danish National Registry of Patients) found that irritable bowel syndrome triggers changes in the gut microbiota that may lead to an increased risk of glaucoma [61]. All these studies demonstrate that dysbiosis of the gut microbiota and their metabolites can play an active role in the development of glaucoma through multiple mechanisms, such as bacterial invasion of the optic nerve and retina, invasion of bacterial products into the optic nerve, and alterations in the immune system.

## 7. Systematic and Neuroinflammation

**Figure 7:**
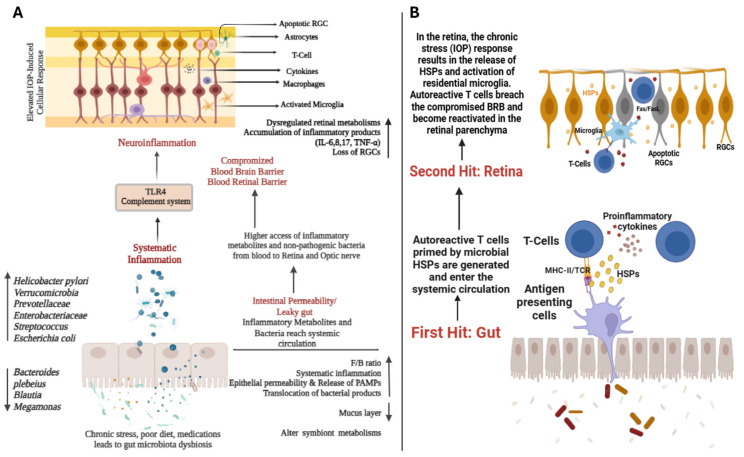
The direct and indirect effects of microbiota dysbiosis on glaucoma. (**A**) Gut microbiota dysbiosis (decrease in total bacterial diversity and richness) induces inflammation (neuroinflammation) and autoimmunity. (**B**) The proposed model for the “dual-hit hypothesis” by Lixiang Wang and Xin Wei [41]. In gut dysbiosis, changes in gut microbiota, weakened gut lining, and bacteria lead to chronic local inflammation. This triggers autoreactive T cells entering the bloodstream (first hit). In the retina, chronic stress activates microglia and releases HSPs. The T cells cross the weakened BRB, reactivating in the retina (second hit), and can induce RGC apoptosis through Fas/FasL interaction. Created using BioRender.com.

Gut dysbiosis results in heightened intestinal permeability, often called leaky gut, which permits harmful agents like inflammatory metabolites and bacteria to infiltrate the systemic circulation, provoking a systemic inflammatory response and contributing to overall inflammation. Prolonged inflammation can harm the BBB and the BRB [20,21]. Consequently, inflammatory metabolites and bacteria can penetrate the retina and optic nerve, triggering and collecting various immune cells, including B cells, T cells, astrocytes, microglia, and macrophages [74,75]. Immune and glial cells, such as astrocytes, microglia, and infiltrating monocytes, are critical in the neuroinflammatory mechanisms associated with glaucoma (Figure 7A) [76]. Based on increasing evidence from animal models, neuroinflammation is an essential process in glaucoma, and there is substantial evidence for its role in the disease [76,77]. While animal models have helped us understand neuroinflammation’s role in glaucoma, expanding the investigation of human subjects and animal models relevant to the condition is essential. The disease’s development initially affects RGCs.

The development and maturation of microglia are significantly influenced by microbiota [78], which typically provide neurotrophic support but can also cause neuron injury [79]. Additionally, high-fat diets have been found to accelerate choroidal neovascularization by modifying gut microbiota, which increases intestinal permeability, chronic inflammation, and pro-inflammatory cytokines and VEGF-A production [34]. Similarly, in the AOH animal model, the antibiotic treatment decreases the activation of retinal microglia and the levels of inflammatory cytokines IL6, Il1beta, and IL10 while reducing retinal miRNA expression, thereby protecting retinal cells [69]. Chen et al. also discovered that modifying the gut microbiota suppresses miR1225p, reducing retinal inflammation and offering neuroprotection [69].

Microbiota-derived bacterial fermentation products, such as SCFAs, have been found to promote microglia homeostasis, as demonstrated in studies [80]. These findings indicate that the quantity and complexity of the microbiota play a key role in microglia function and that a diverse microbiota could help regulate microglia maturation and function. Consequently, complex microbiota may offer some relief for the functional impairment of microglia [21]. Molecular pathways, including the complement cascade, toll-like receptors, and TNF-α pathway, have a critical role in regulating neuroinflammation and have been linked to the development of glaucoma in human and animal models, as evidenced by studies [3,81]. Overall, gut dysbiosis can cause the spread of proinflammatory metabolites and bacteria systemically through a leaky gut, resulting in systemic inflammation. This process may lead to changes in the retinal barriers, increasing access to harmful bacteria and metabolites at the retina and optic nerve levels. As a result, local inflammation (neuroinflammation) may worsen, leading to the death of RGCs and neurodegeneration.

## 8. Autoimmunity

The involvement of autoimmunity and imbalanced immune responses in the early stages of glaucoma is due to the emergence of autoantibodies, shifts in T-cell subpopulations, and the activation of retinal innate immunity, all of which play a pivotal role in disease pathogenesis [55]. Recently, there has been renewed attention to the effects of microbiota on glaucoma pathogenesis, with a focus on autoimmune or inflammatory components. The mechanism behind immune response initiation in glaucoma is not yet fully understood. Still, evidence suggests that it may involve the recall of immune memory rather than the induction of a glaucoma-specific antigen [82].

Evidence suggests that immune responses in glaucoma may be activated by T cells directed against heat shock proteins (HSPs) [83]. The HSPs, which play a significant role in signaling and stress response in gut microbiota-mediated immune responses, interest researchers [2]. A study conducted in 1998 found autoantibodies against tiny HSPs in the serum of patients with POAG [84], indicating the involvement of immunological reactions to HSPs in the disease [85,86]. Further research showed that glaucoma patients have higher levels of anti-heat shock protein autoantibodies, including antibody levels to HSP-27, and decreased antibody reactivity against enolase [87]. The detection of antibodies directed against HSPs in glaucoma patients suggests that individuals may develop immune responses against HSPs of gut bacteria at some point in their lives [2]. Chen et al. conducted a study that found that CD4+ T-lymphocytes, recognizing HSPs, are linked to the immune mechanism involved in the pathogenesis of neural damage in glaucoma. The study also found that bacterial HSPs can cross-react with mouse and human HSPs. Moreover, elevated IOP values were found to cause the influx of CD4+ T-cells into the retina, which was identified as responsible for the extended phase of neurodegeneration in glaucomatous disease, as shown in germ-free mice [51].

The multifaceted nature of autoreactive T cells and their neurotoxic or neuroprotective ability highlight the complexity of neuroinflammation in glaucoma progression. Imbalances in T cell/microglia interactions are believed to be the underlying cause of the disease [88]. The neuroinflammatory environment, abundant with pro-inflammatory cytokines, facilitates glia-T lymphocyte interactions, contributing to an increased immune response [89]. Moreover, glaucoma gene and protein expression studies have revealed the upregulation of several chemotaxis and adhesion molecules necessary for the glia-T cell interaction [90]. Activated T cells have been shown to infiltrate the injury site in animal models and produce neurotoxic inflammatory outcomes that damage RGCs. Additionally, recent studies have identified an imbalance in the regulatory subset of T cells (Treg). This suggests a lack of efficient T-cell suppression in some glaucoma patients [91].

According to Chen et al., in an in vivo study, glaucoma develops when bacteria-primed CD4+ T lymphocytes traverse the BRB, interact with HSP-expressing RGCs, and induce neurodegeneration. The migration of CD4+ T cells into RGCs correlates with elevated IOP before the degeneration of RGCs and their axons. A higher prevalence of HSP27-specific and HSP60-specific T cells was observed [51]. Further findings indicated that these T lymphocytes were specific to HspB1, and bacterial HSP-specific memory T cells that infiltrated the retina via a disrupted BRB were activated by local HSPs. Additionally, it was observed that these T-cell responses and the resulting neuronal damage in glaucoma did not occur in mice raised without gut microbiota, suggesting that the gut microbiome may play a role in promoting glaucoma-related processes [51].

Wang et al. reviewed the relationship between peripherally activated T cells and glaucoma. They proposed a “Dual-hit hypothesis”, suggesting that the first hit occurs due to gut dysbiosis and changes in gut microbiota, leading to chronic local inflammation, autoreactive T-cell generation, and microbial HSP priming. In the second hit, chronic retinal stress causes the release of HSPs and activation of resident microglia, allowing the autoreactive T cells to cross the BRB and reactivate in the retinal parenchyma. The T cells induce RGCs apoptosis through Fas/FasL interactions. The local proinflammatory microenvironment facilitates T-cell infiltration by producing cytokines, selectins, and chemokines, involving more microglia and dendritic cells; see Figure 7B [55].

## 9. Modulating Gut Microbiota: Emerging Therapeutic Strategies for Glaucoma

Probiotic regimens are a popular dietary intervention due to their noninvasive properties and ability to improve gut health, which in turn helps maintain a healthy immune system. Probiotics are believed to modulate immune responses, protect against physiological stress, suppress pathogen invasion, and improve the barrier function of the gut epithelium by modulating the gut microbiota [92,93]. Research indicates that *lactobacilli* can suppress TNF-α expression, reducing chronic inflammation, reducing neutrophil extracellular traps, and potentially helping to treat neurological and autoimmune diseases [94,95]. A probiotic combination of *lactobacillus gasseri KS-13*, *Bifidobacterium bifidum G9-1*, and *B. longum MM-2* has been shown to improve rhino conjunctivitis by increasing serum total IgE and Treg percentage [96], also enhancing the function of RPE cells under chronic inflammatory stress [97]. Similarly, an eye drop probiotic formula containing *Saccharomyces boulardii* and *Lactobacillus rhamnosus* was effective against keratoconjunctivitis [98]. The IRT-5 regimen consisting of *Lactobacillus casei*, *Lactobacillus acidophilus*, *Lactobacillus reuteri*, *Bifidobacterium bifidum*, and *Streptococcus thermophilus* has been shown to reduce autoimmunity in the eye [99]. Supplementation of *Bifidobacterium* enhances RGC survival and optic nerve regeneration by inhibiting microglia activation while promoting Müller cell activity. This process is associated with reduced inflammatory cytokine levels, increased neurotrophic factors, and activation of the retinal ERK/Fos signaling pathway. This study suggests that Bifidobacterium-induced changes in gut flora contribute to RGC survival by suppressing microglia activation and stimulating Müller cells, leading to secondary regulation of inflammation and neurotrophic support [100].

Fecal microbiota transplant (FMT) is another therapeutic approach that aims to restore the natural balance of gut bacteria in patients by transferring healthy donor feces. FMT and probiotics have been shown to repopulate target gut microbiota and reduce the severity of uveitis [101]. Furthermore, FMT has been found to reverse disruptions to the gut barrier and inflammation affecting the retina, potentially improving age-related macular degeneration. Trials are underway to investigate the potential benefits of FMT on psoriatic and rheumatoid arthritis, both known to be associated with uveitis [102]. In a recent study, FMT of primary open-angle glaucoma POAG patients increased the expression of miR-122-5p in the mouse retina. In contrast, the FMT of healthy subjects decreased the expression [69].

**Figure 8:**
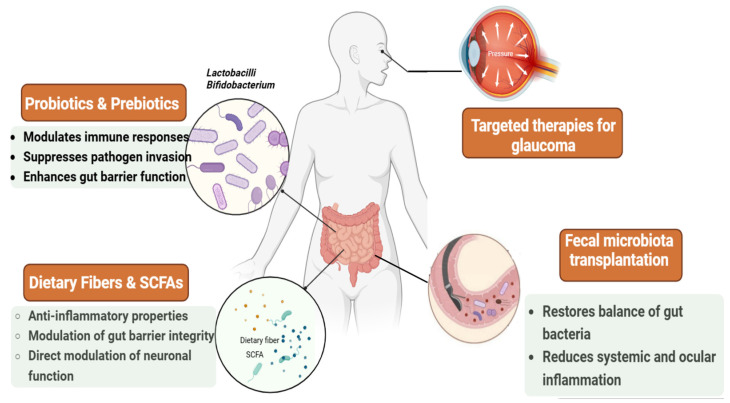
Gut microbiota modulation as a potential therapy for glaucoma. The figure shows the advantages of gut microbiota-modifying therapies. Probiotics and prebiotics, such as Lactobacillus and Bifidobacterium, help regulate the immune response and reduce inflammation. Diets high in fermented foods and fiber introduce probiotics and SCFAs positively impact the gut microbiota. Fecal microbiota transplantation from healthy donors has been shown to reduce inflammation in diseases beyond the gut, suggesting it could significantly treat glaucoma. Further research on these therapies in the context of glaucoma could lead to more therapeutic strategies. Created using BioRender.com.

SCFAs arise in the complex connections between the gut microbiota and the central nervous system, emerging as potential candidates for therapeutic interventions to mitigate neurodegenerative diseases [103]. Beyond their known anti-inflammatory role, SCFAs induce the regulation of gut barrier integrity, immune response modulation, and direct modulation of neuronal function [104]. A recent study found that administering SCFAs to retinal astrocytes reduces intraocular inflammation triggered by lipopolysaccharide. This reduction was linked to lower production of cytokines and chemokines, which enhanced the potential for T-cell activation [105]. SCFAs have efficiently ameliorated the pathophysiology of middle cerebral artery occlusion in rodent models, suggesting promising therapeutic prospects [23]. Supplementation with butyric acid, a type of SCFAs, has been shown to induce alterations in gut microbiota composition, consequently helping to restore compromised intestinal barrier function [106]. Investigations into gut microbiota have revealed specific SCFA-producing capabilities of certain bacterial strains. For instance, *Parabacteroides distasonis* has been identified as a source of hexanoic acid, while *Megasphaera massiliensis* secretes a combination of butyric acid, valeric acid, and hexanoic acid [107]. These findings emphasize the potential significance of these bacteria in modulating oxidative stress and inflammation, critical factors in neuroinflammation, and neurodegenerative diseases; see Figure 8.

## 10. Conclusions

Our comprehensive review and bibliometric analysis highlight the growing interest in the association between gut microbiota and glaucoma. The microbiota–gut–retina axis has emerged as a significant area of research, offering a novel perspective on glaucoma pathogenesis. Our study identifies the USA and China as the top contributors, with the highest number of articles and serving as key research hubs. Leading journals such as *Experimental Eye Research* and *Investigative Ophthalmology & Visual Science* were found to be leading in generating high-quality publications. The most frequently occurring keywords in this analysis included “glaucoma”, “microbiota”, “gut microbiota”, “inflammation”, “gut-retina axis”, and “probiotics”. Additionally, our study summarizes the possible ways in which gut microbiota dysbiosis, systemic and neuroinflammation, and autoimmune mechanisms contribute to glaucomatous pathogenesis. However, despite substantial studies exploring the potential links between gut microbiota and ocular health, the molecular mechanisms underlying this relationship remain largely unexplored. To advance the understanding of glaucoma pathogenesis, future research should prioritize mechanistic studies to elucidate the specific pathways by which gut microbiota influence glaucoma development and progression. Techniques such as metagenomics, metabolomics, and epigenomics offer promising approaches for identifying how specific microbiota compositions and their metabolic byproducts impact retinal health. Furthermore, targeted probiotic and prebiotic therapies present a novel therapeutic strategy for glaucoma. Such interventions could modulate the gut microbiota to reduce systemic and neuroinflammation, enhance retinal barrier function, and prevent neurodegeneration. By integrating these new approaches, future studies could move beyond correlational findings and provide insights into how modulating the gut microbiota may offer innovative, personalized treatments for glaucoma. These interventions have the potential to not only slow disease progression but also improve overall patient outcomes, offering a new advancement in glaucoma therapy.

## 11. Limitations

This study has some limitations. One considerable limitation is the potential for data source bias, as bibliometric analysis depends merely on available publications. This leads to the possibility of publication bias, where studies with significant findings may be overrepresented, while inconclusive results are often underreported. Additionally, since the analysis is based on existing literature and does not include new experimental data, there is a lack of direct experimental validation for the findings or hypotheses discussed. Furthermore, the field of gut microbiota and glaucoma is relatively new. As research evolves, emerging studies may further refine or challenge the current understanding of the role of gut microbiota in glaucoma pathogenesis.

## Figures and Tables

**Table 1:** Search topic of this study.

Search Term	Search Query
#1	Topic = (gut microbiota OR gut microbiotas OR gut microbiome OR gut microbiomes OR gut microflora OR gut flora OR gastrointestinal microbiota OR gastrointestinal microbiotas OR gastrointestinal microbiome OR gastrointestinal microbiomes OR gastrointestinal microbial community OR gastrointestinal microbial communities OR gastrointestinal bacteria OR gastrointestinal flora OR gastrointestinal microflora OR intestinal microbiota OR intestinal microbiotas OR intestinal microbiome OR intestinal microbiomes OR intestinal microflora OR intestinal flora OR intestinal bacteria OR gut bacteria OR gastric microbiome OR gastric microbiomes OR fecal microbiota OR fecal microbiomas OR fecal microbiome OR fecal microbiomes OR fecal bacteria OR fecal flora OR fecal microflora OR enteric bacteria OR microbiome, gastrointestinal OR microbiome, gut OR microflora, gut OR microbiota, gut OR flora, gastrointestinal OR flora, gut OR microbiota, gastrointestinal OR microbial community, gastrointestinal OR microflora, gastrointestinal OR microbiome, gastric OR microbiome, intestinal OR microflora, intestinal OR flora, intestinal OR bacteria, enteric OR probiotic * OR prebiotic * OR synbiotic * OR postbiotic *)
#2	Topic = (glaucoma* OR hydrophthalmos OR ocular hypertension* OR oculus caesius OR choroiditis serosa OR glaucedo OR buphthalmos)
#3	#1 AND #2

* It is a wildcard used to find plural.

**Table 2:** Top 10 prolific institutions.

Rank	Institution	Documents	Total Citations	Average Publication Year	Average Citations	Links
1	Sun Yat Sen University	5	152	2020.8	30.4	12
2	Harvard Medical School	3	26	2021.3	8.7	13
3	University of Chicago	2	47	2020.0	23.5	0
4	Fudan University	2	31	2021.0	15.5	4
5	Aristotle University of Thessaloniki	2	29	2020.5	14.5	6
6	Sichuan University	2	20	2021.5	10.0	3
7	Tohoku University	2	14	2022.0	7.0	1
8	Central South University	2	8	2023.5	4.0	2
9	University of Sydney	2	6	2022.0	3.0	14
10	University of Oxford	2	1	2023.5	0.5	9

**Table 3:** Top five prolific journals and co-cited journals.

Rank	Journal	Documents	Total Citations	Average Publication Year	Average Citations	Impact Factor	Co-Cited Journal	Co-Citations	Impact Factor
1	Experimental Eye Research	3	74	2022.0	24.7	3.0	Investigative Ophthalmology & Visual Science	193	5.0
2	International Journal of Molecular Sciences	3	22	2023.3	7.3	4.9	PloS One	113	2.9
3	International Ophthalmology	3	20	2022.3	6.7	1.4	Scientific Reports	102	3.8
4	Frontiers in Cellular and Infection Microbiology	2	39	2021.5	19.5	4.6	Nutrients	87	4.8
5	Medicina-Lithuania	2	6	2023.5	3.0	2.4	Nature	84	50.5

## Data Availability

The corresponding author can provide raw and processed data upon request.
